# Association of Depressive Symptoms With Incident Cardiovascular Diseases in Middle-Aged and Older Chinese Adults

**DOI:** 10.1001/jamanetworkopen.2019.16591

**Published:** 2019-12-04

**Authors:** Haibin Li, Deqiang Zheng, Zhiwei Li, Zhiyuan Wu, Wei Feng, Xue Cao, Jiaxin Wang, Qi Gao, Xia Li, Wei Wang, Brian J. Hall, Yu-Tao Xiang, Xiuhua Guo

**Affiliations:** 1 Department of Epidemiology and Health Statistics, Capital Medical University School of Public Health, Beijing, China; 2 Beijing Municipal Key Laboratory of Clinical Epidemiology, Capital Medical University, Beijing, China; 3 Department of Mathematics and Statistics, La Trobe University, Melbourne, Victoria, Australia; 4 Global Health and Genomics, Edith Cowan University School of Medical Health Sciences, Perth, Western Australia, Australia; 5 Global and Community Mental Health Research Group, Department of Psychology, University of Macau, Macao SAR, China; 6 Health, Behavior, and Society, Johns Hopkins Bloomberg School of Public Health, Baltimore, Maryland; 7 Unit of Psychiatry, Institute of Translational Medicine, Faculty of Health Sciences, University of Macau, Macao SAR, China; 8 Center for Cognition and Brain Sciences, University of Macau, Macao SAR, China

## Abstract

**Importance:**

The prevalence of depressive symptoms among older adults has become an increasingly important public health priority. Elevated depressive symptoms are well documented among elderly people with cardiovascular disease (CVD), but studies conducted among Chinese adults are scarce.

**Objective:**

To estimate the association between depressive symptoms and incident CVD among middle-aged and older Chinese adults.

**Design, Setting, and Participants:**

The China Health and Retirement Longitudinal Study is an ongoing nationally representative prospective cohort study that was initiated in 2011. This cohort study included 12 417 middle-aged and older Chinese adults without heart disease and stroke at baseline. Statistical analysis was conducted from April 25, 2018, to December 13, 2018.

**Exposure:**

Depressive symptoms were assessed using the validated 10-item of Center for Epidemiologic Studies Depression Scale.

**Main Outcomes and Measures:**

Incident CVD (ie, self-reported physician-diagnosed heart disease and stroke combined) was followed-up from June 1, 2011, to June 31, 2015. The Center for Epidemiologic Studies Depression Scale total score ranges from 0 to 30, with a score of 12 or more indicating elevated depressive symptoms.

**Results:**

Of the 12 417 participants (mean [SD] age at baseline, 58.40 [9.51] years), 6113 (49.2%) were men. During the 4 years of follow-up, 1088 incident CVD cases were identified. Elevated depressive symptoms were independently associated with an increased CVD risk (adjusted hazard ratio, 1.39; 95% CI, 1.22-1.58) after adjusting for age, sex, residence, marital status, educational level, smoking status, drinking status, systolic blood pressure, and body mass index; history of diabetes, hypertension, dyslipidemia, and chronic kidney disease; and use of hypertension medications, diabetes medications, and lipid-lowering therapy. Of the 10 individual depressive symptoms measured by the Center for Epidemiologic Studies Depression Scale, only 2 symptoms, restless sleep (adjusted hazard ratio, 1.21; 95% CI, 1.06-1.39) and loneliness (adjusted hazard ratio, 1.21; 95% CI, 1.02-1.44), were significantly associated with incident CVD.

**Conclusions and Relevance:**

Elevated depressive symptoms overall and 2 individual symptoms (restless sleep and loneliness) were significantly associated with incident CVD among middle-aged and older Chinese adults.

## Key Points

**Question:**

Are depressive symptoms associated with incident cardiovascular disease among middle-aged and older Chinese adults?

**Findings:**

In this cohort study of 12 417 Chinese adults, participants with depressive symptoms at baseline had higher incident rates of cardiovascular disease compared with those without such symptoms. Elevated depressive symptoms as a whole and 2 individual symptoms (restless sleep and loneliness) were significantly associated with increased risk of cardiovascular disease after adjusting for potential confounders.

**Meaning:**

This study suggests that depressive symptoms, particularly restless sleep and loneliness, may be associated with incident cardiovascular disease among middle-aged and older Chinese adults.

## Teaser

This cohort study examines the association between depressive symptoms and incident cardiovascular disease among middle-aged and older Chinese adults.

## Introduction

Depressive symptoms are common among middle-aged and older adults.^1,2^ China, like much of Asia, is experiencing an increase in older adults. The prevalence of depressive symptoms in older adults has become an increasingly important public health priority.^3,4,5^ A national survey in China^6^ showed that approximately 30% of men and 43% of women aged 45 years and older experienced depressive symptoms. Epidemiological studies^7,8,9^ have found that depressive symptoms are associated with a range of negative health outcomes, such as coronary heart diseases, stroke, and all-cause mortality.

Some studies^10,11,12^ found that both the history and new onset of depressive symptoms were associated with a series of cardiovascular events, such as angina, myocardial infarction, stroke, heart failure, and coronary death. A meta-analysis^13^ of 24 prospective cohort studies found that depressive symptoms could be associated with a 30% excess risk for coronary heart disease. The association between depression and incident cardiovascular disease (CVD) may vary across different depression subtypes^14^ and may be bidirectional; for example, depressive symptoms are associated with an increased risk of CVD,^13^ whereas cardiovascular risk factors also are associated with depression or depressive symptoms.^15,16^ Therefore, to reduce the risk of CVD, it is important to understand its association with depressive symptoms.

Depressive symptoms are usually assessed using validated rating scales with established cutoffs, such as the Center for Epidemiologic Studies Depression Scale (CES-D)^17^ and the Geriatric Depression Scale.^18^ Although these scales cannot be used to establish the diagnosis of major depression, they are widely used in both research and daily practice, and most confirmatory factor analysis studies^19^ support 2 clusters of symptoms: emotional or affective (eg, felt depressed, happy, or lonely) and somatic symptoms (eg, fatigue, appetite, or sleep). Most previous studies^7,12,20,21,22^ on the association between depressive symptoms and CVD risk only analyzed the presence of depressive symptoms as a binary variable (eg, depressed or not) or used its total score, with the assumption that all depressive symptoms are equally good severity indicators,^23^ despite the lack of evidence to support this.

To date, the contribution of individual depressive symptoms to incident CVD is still unknown, which gives us the impetus to examine the association between specific depressive symptoms and incident CVD among middle-aged and older adults in China on the basis of the China Health and Retirement Longitudinal Study (CHARLS). We hypothesized that elevated depressive symptoms and certain specific depressive symptoms would be associated with increased risk of CVD.

## Methods

### Study Population

This cohort study was a secondary analysis of the data set of the CHARLS, which is an ongoing nationally representative cohort study.^24^ Details of the study design have been described elsewhere.^24,25^ In brief, a total of 17 708 participants in 10 257 households were recruited from 150 counties or districts and 450 villages within 28 provinces in China between June 2011 and March 2012, using the multistage stratified probability-proportional-to-size sampling technique. All participants underwent an assessment using a standardized questionnaire to collect data on sociodemographic and lifestyle factors and health-related information. The response rate of the baseline survey was 80.5%. All participants were followed up every 2 years after the baseline survey.

The CHARLS study was approved by the institutional review board of Peking University. Written informed consent was obtained from all participants. This study was conducted following the Strengthening the Reporting of Observational Studies in Epidemiology (STROBE) reporting guideline.^26^

### Assessment of Depressive Symptoms

In the baseline survey of the CHARLS, depressive symptoms were measured using the CES-D short form,^17^ which is a widely used self-report measure on depressive symptoms in population-based studies. The CES-D short form consists of 10 items: (1) bothered by little things, (2) had trouble concentrating, (3) felt depressed, (4) everything was an effort, (5) felt hopeful, (6) felt fearful, (7) sleep was restless, (8) felt happy, (9) felt lonely, and (10) could not get going. Depressive symptoms in the past week were measured from 0 (rarely or none of the time [<1 day]) to 3 (most or all of the time [5-7 days]). Before summing item scores, items 5 and 8 need to be reverse scored. The CES-D total score varies from 0 to 30, with a higher score indicating more depressive symptoms. The CES-D short form has excellent psychometric properties among Chinese older adults.^27^ A total score of 12 or higher was used as the cutoff for having elevated depressive symptoms.^27^

### Ascertainment of Incident CVD Events

The study outcome was incident CVD events. In accordance with previous studies,^28,29^ incident CVD events were assessed by the following standardized questions: “Have you been told by a doctor that you have been diagnosed with a heart attack, coronary heart disease, angina, congestive heart failure, or other heart problems?” or “Have you been told by a doctor that you have been diagnosed with a stroke?” Participants who reported heart disease or stroke during the follow-up period were defined as having incident CVD. The date of CVD diagnosis was recorded as being between the date of the last interview and that of the interview reporting an incident CVD.^28,29^

### Covariates

At baseline, trained interviewers collected information on sociodemographic status and health-related factors using a structured questionnaire, including age, sex, living residence, marital status, and educational level. Educational level was classified as no formal education, primary school, middle or high school, and college or above. Health-related factors included self-reported smoking and drinking status (never, former, or current), self-reported physician-diagnosed medical conditions (diabetes, hypertension, dyslipidemia, and chronic kidney disease), and use of medications for diabetes, hypertension, and dyslipidemia. Marital status was classified into 2 groups: married and other marital status (never married, separated, divorced, and widowed). Height, weight, and blood pressure were measured by a trained nurse. Body mass index was calculated as weight in kilograms divided by height in meters squared.

A subcohort of 8696 CHARLS participants underwent metabolic examinations, including fasting plasma glucose, total cholesterol, triglycerides, high-density lipoprotein cholesterol, low-density lipoprotein cholesterol, high-sensitivity C-reactive protein, and serum creatinine. The estimated glomerular filtration rate was calculated using the Chronic Kidney Disease Epidemiology Collaboration’s 2009 creatinine equation.^30^

### Statistical Analysis

Statistical analysis was conducted from April 25, 2018, to December 13, 2018. Data were described as means and SDs for normally distributed continuous variables, and as medians and interquartile ranges for nonnormally distributed continuous variables. Frequency with percentage was used to describe categorical variables. Baseline characteristics are summarized according to depressive symptoms and compared between participants with and without elevated depressive symptoms using the χ^2^ test, analysis of variance, or Mann-Whitney *U* test, as appropriate. Eighteen percent (2235 of 12 417) of total data items were missing, were assumed to be missing at random, and, thus, were imputed with the multiple imputation of chained equations method using the baseline characteristics. We created 10 imputed data sets and pooled the results using the Stata statistical software version 15.1 (StataCorp) command “mi estimate.”

We computed the person-time of follow-up for each participant from the date of the 2011 to 2012 survey (baseline) to the dates of the CVD diagnosis, death, loss to follow-up (605 of 12 417 [4.9%]), or the end of follow-up (June 31, 2015), whichever came first. Incidence rates of CVD events per 1000 person-years were calculated by depressive symptoms. To examine the association between depressive symptoms and incident CVD events, Cox proportional hazards models were used to calculate hazard ratios (HRs) with 95% CIs. Three models were estimated: in model 1, age and sex were adjusted; in model 2, age, sex, residence, marital status, educational level, smoking status, and drinking status were adjusted; and in model 3, the variables in model 2 plus history of diabetes, hypertension, dyslipidemia, and chronic kidney disease; systolic blood pressure and body mass index; and use of hypertension medications, diabetes medications, and lipid-lowering therapy were adjusted. To examine the association between specific depressive symptoms (eg, CES-D individual items) and incident CVD events, using the method of Jokela et al,^31^ we coded the items as dichotomous variables by defining the responses as occasionally or a moderate amount of time (3-4 days) and all of the time (5-7 days) as having the specific symptoms. All 10 items were entered simultaneously in model 3.

To further examine the association between the severity of depressive symptoms and incident CVD events, scores of depressive symptoms were split into quintiles and then were included in Cox proportional hazards models with quintile 1 as the reference group. In addition, we explored the potential nonlinear associations using 3-knotted restricted cubic spline regression. Subgroup analyses were conducted to examine whether the potential association between depressive symptoms and CVD events was moderated by the following demographic and clinical characteristics: age, sex, residence, marital status, educational level, smoking status, drinking status, diabetes (defined as fasting plasma glucose ≥126 mg/dL [to convert to millimoles per liter, multiply by 0.0555], current use of antidiabetic medication, or self-reported history of diabetes), hypertension (defined as systolic blood pressure ≥140 mm Hg, diastolic blood pressure ≥90 mm Hg, current use of the antihypertensive medication, or self-reported history of hypertension), dyslipidemia (defined as total cholesterol ≥240 mg/dL [to convert to millimoles per liter, multiply by 0.0259], triglycerides ≥150 mg/dL, low-density lipoprotein cholesterol ≥160 mg/dL, high-density lipoprotein cholesterol <40 mg/dL, current use of lipid-lowering medication, or self-reported history of dyslipidemia), chronic kidney disease (defined as estimated glomerular filtration rate <60 mL/min/1.73 m^2^ or self-reported history of chronic kidney disease), and body mass index. *P* values for interaction were evaluated using interaction terms and likelihood ratio tests.

Three sensitivity analyses were conducted as follows: (1) further adjusting for metabolic biomarkers in model 3 in the sample of 8696 participants who underwent metabolic examinations; (2) repeating all analyses using the complete data set (10 186 participants) without multiple imputations; and (3) using the Fine and Gray competing risk model^32^ to account for competing risks due to mortality. Two-sided *P* < .05 was considered as statistically significant. All analyses were performed using Stata statistical software version 15.1 (StataCorp) and R statistical software version 3.6.1 (R Foundation).

## Results

Of the 17 708 CHARLS participants at study baseline, we excluded 1841 individuals younger than 45 years, 2789 with heart disease or stroke at baseline, 1878 with incomplete information on depressive symptoms, and 140 with no answers for the questions on the physician-diagnosis CVD during follow-up. Finally, 12 417 participants were included for analysis, and 8696 (70.0%) of them provided blood samples at baseline. A comparison of baseline characteristics between participants included and those who were not included in the analysis is shown in eTable 1 in the Supplement.

A total of 12 417 adults were included in the analyses. The mean (SD) age at baseline was 58.40 (9.51) years; 6113 (49.2%) of the participants were men and 6304 (50.8%) were women. Table 1 shows the characteristics of the participants. At baseline, 3223 participants (26.0%) had elevated depressive symptoms (CES-D total score ≥12). Univariate analysis revealed that compared with those without depressive symptoms, participants with depressive symptoms were more likely to be older (mean [SD] age, 60.10 [9.85] vs 57.81 [9.32]; difference, 2.29 years; 95% CI, 19.91 to 2.67 years; *P* < .001), be female (60.9% vs 47.2%; difference, 13.7%; 95% CI, 11.7% to 15.6%;* P* < .001), live in a rural setting (rural residence, 71.9% vs 57.2%; difference, 14.7%; 95% CI, 12.8% to 16.5%;* P* < .001), be unmarried (23.8% vs 14.2%; difference 9.6%; 95% CI, 8.0% to 11.2%;* P* < .001), have no formal education (59.1% vs 39.2%; difference, 19.9%; 95% CI, 17.9% to 21.8%;* P* < .001), be never smokers (64.7% vs 58.0%; difference, 6.7%; 95% CI, 4.7% to 8.6%;* P* < .001), be never drinkers (63.1% vs 56.2%; difference, 6.9%; 95% CI, 5.0% to 8.9%;* P* < .001), have a history of hypertension (24.6% vs 21.6%; difference, 3.0%; 95% CI, 1.3% to 4.7%;* P* < .001) and chronic kidney disease (8.0% vs 4.1%; difference, 3.9%; 95% CI, 2.9% to 5.0%; *P* < .001), use medications for diabetes (3.8% vs 3.0%; difference, 0.8%; 95% CI, 0.1% to 1.6%;* P* = .02) and hypertension (17.3% vs 15.2%; difference, 2.1%; 95% CI, 0.6% to 3.6%;* P* = .01), and have lower diastolic blood pressure (mean [SD], 75.02 [12.27] mm Hg vs 76.20 [12.07] mm Hg; difference, −1.18 mm Hg; 95% CI, −1.70 to −0.66 mm Hg; *P* < .001), body mass index (mean [SD], 22.76 [3.78] vs 23.55 [3.86]; difference, −0.78; 95% CI, −0.95 to −0.62; *P* < .001), and estimated glomerular filtration rate (mean [SD], 92.25 [14.88] mL/min/1.73 m^2^ vs 93.11 [14.67] mL/min/1.73 m^2^; difference, −0.87 mL/min/1.73 m^2^; 95% CI, −1.57 to −0.16 mL/min/1.73 m^2^; *P* < .02) but higher high-density lipoprotein cholesterol (mean [SD], 52.53 [15.75] mg/dL vs 50.84 [15.20] mg/dL; difference, 1.68 mg/dL; 95% CI, 0.95 to 2.42 mg/dL; *P* < .001).

**Table 1.** Baseline Characteristics of 12 417 Participants According to Depressive Symptoms Status

Characteristics	Participants, No. (%)			*P* Value^b^
	Total Sample (N = 12 417)	Depressive Symptoms		
		No (n = 9194)	Yes (n = 3223)^a^	
Age, mean (SD), y	58.40 (9.51)	57.81 (9.32)	60.10 (9.85)	<.001
Men	6113 (49.2)	4852 (52.8)	1261 (39.1)	<.001
Rural residence	7577 (61.0)	5260 (57.2)	2317 (71.9)	<.001
Married	10 344 (83.3)	7888 (85.8)	2456 (76.2)	<.001
Educational level^c^				
No formal education	5508 (44.4)	3604 (39.2)	1904 (59.1)	<.001
Primary school	2695 (21.7)	2032 (22.1)	663 (20.6)	
Middle or high school	3648 (29.4)	3032 (33.0)	616 (19.1)	
College or above	565 (4.6)	525 (5.7)	40 (1.2)	
Smoking status^c^				
Never	7414 (59.7)	5330 (58.0)	2084 (64.7)	<.001
Former	1011 (8.1)	786 (8.6)	225 (7.0)	
Current	3989 (32.1)	3075 (33.5)	914 (28.4)	
Drinking status^c^				
Never	7196 (58.0)	5162 (56.2)	2034 (63.1)	<.001
Former	953 (7.7)	660 (7.2)	293 (9.1)	
Current	4265 (34.4)	3370 (36.7)	895 (27.8)	
History of comorbidities				
Diabetes^c^	628 (5.1)	449 (4.9)	179 (5.6)	.13
Hypertension^c^	2768 (22.4)	1980 (21.6)	788 (24.6)	<.001
Dyslipidemia^c^	960 (7.9)	729 (8.0)	231 (7.3)	.18
Chronic kidney disease^c^	629 (5.1)	372 (4.1)	257 (8.0)	<.001
History of medication use				
Diabetes medications^c^	392 (3.2)	270 (3.0)	122 (3.8)	.02
Hypertension medications^c^	1946 (15.7)	1391 (15.2)	555 (17.3)	.01
Lipid-lowering therapy^c^	464 (3.8)	345 (3.8)	119 (3.8)	.91
Blood pressure, mean (SD), mm Hg^c^				
Systolic	130.15 (21.31)	130.18 (21.03)	130.07 (22.07)	.81
Diastolic	75.89 (12.13)	76.20 (12.07)	75.02 (12.27)	<.001
Body mass index, mean (SD)^c^^,^^d^	23.34 (3.85)	23.55 (3.86)	22.76 (3.78)	<.001
Metabolic biomarkers^e^				
Total cholesterol, mean (SD), mg/dL	193.02 (38.43)	192.82 (38.78)	193.58 (37.43)	.42
Triglycerides, median (IQR), mg/dL	104.43 (78.76)	105.32 (80.54)	102.66 (73.45)	.06
Cholesterol, mean (SD), mg/dL				
High-density lipoprotein	51.29 (15.37)	50.84 (15.20)	52.53 (15.75)	<.001
Low-density lipoprotein	116.04 (34.78)	115.78 (34.91)	116.73 (34.45)	.26
Fasting plasma glucose, mean (SD), mg/dL	109.63 (36.51)	109.63 (35.81)	109.63 (38.38)	>.99
Estimated glomerular filtration rate, mean (SD), mL/min/1.73 m^2^	92.88 (14.73)	93.11 (14.67)	92.25 (14.88)	.02
High-sensitivity C-reactive protein, median (IQR), mg/L	1.02 (1.59)	1.04 (1.55)	0.98 (1.69)	.89

Abbreviation: IQR, interquartile range (75th quartile minus 25th quartile).

SI conversion factors: To convert total cholesterol, triglycerides, high-density lipoprotein cholesterol, and low-density lipoprotein cholesterol to mmol/L, multiply by 0.0259; to convert fasting plasma glucose to mmol/L, multiply by 0.0555; and to convert high-sensitivity C-reactive protein to nmol/L, multiply by 9.524.

^a^ Defined as a score of 12 or greater on the 10-item Center for Epidemiologic Studies Depression Scale.

^b^ *P* value was based on χ^2^ or analysis of variance or Mann-Whitney *U* test where appropriate.

^c^ Missing data: 1 for educational level, 3 for smoking, 3 for drinking, 90 for diabetes, 56 for hypertension, 200 for dyslipidemia, 40 for chronic kidney disease, 90 for diabetes medications, 56 for hypertension medications, 200 for lipid-lowering therapy, 1835 for systolic blood pressure, 1834 for diastolic blood pressure, and 1922 for body mass index.

^d^ Calculated as weight in kilograms divided by height in meters squared.

^e^ Measured in subpopulation of 8696 participants.

**Figure 1.**
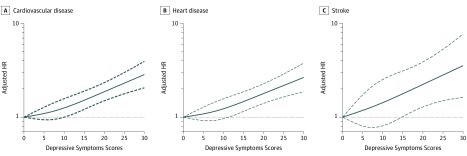
Adjusted Hazard Ratios (HRs) of Cardiovascular Disease Events Risk, According to Depressive Symptoms Scores Graphs show HRs for cardiovascular disease (A), heart disease (B), and stroke (C) adjusted for age, sex, residence, marital status, educational level, smoking status, drinking status, systolic blood pressure, and body mass index; history of diabetes, hypertension, dyslipidemia, and chronic kidney disease; and use of hypertension medications, diabetes medications, and lipid-lowering therapy. Data were fitted by a restricted cubic spline Cox proportional hazards regression model. The depressive symptoms score ranges from 0 to 30, with the highest score representing the lowest risk of depressive symptoms. Solid lines indicate HRs, and dashed lines indicate 95% CIs.

During the follow-up period between 2011 and 2015, 1088 participants experienced incident CVD (heart disease, 929 cases; stroke, 190 cases). The incidence rate of CVD was 29.18 per 1000 person-years among participants with elevated depressive symptoms and 20.55 per 1000 person-years among participants without elevated depressive symptoms. Table 2 shows the associations between depressive symptoms and incident CVD events. After adjusting for potential confounders (in model 3), the presence of elevated baseline depressive symptoms was independently associated with a 39.0% increased risk of incident CVD (CVD: adjusted HR, 1.39; 95% CI, 1.22-1.58; heart disease: adjusted HR, 1.36; 95% CI, 1.18-1.57; stroke: adjusted HR, 1.45; 95% CI, 1.06-1.99). Similar results were found when modeling the total CES-D scores as quintiles (Table 2). After adjusting for confounders, by comparing quintile 5 with quintile 1, the adjusted HRs were 1.75 (95% CI, 1.45-2.11) for incident CVD, 1.73 (95% CI, 1.42-2.12) for heart disease, and 1.69 (1.09-2.64) for stroke. A linear and positive association between the CES-D total score and risk of incident CVD events using restricted cubic spline regression was also found (for nonlinearity, *P* = .30 for CVD, *P* = .40 for heart disease, and *P* = .82 for stroke) (Figure 1).

**Table 2.** Incidence of Cardiovascular Diseases According to Depressive Symptoms Status

Outcome	Cases, No.	Incidence Rate, per 1000 Person-Years	HR (95% CI)		
			Model 1^a^	Model 2^b^	Model 3^c^
Cardiovascular disease					
Depressive symptoms status					
No symptoms	727	20.55	1 [Reference]	1 [Reference]	1 [Reference]
Symptoms^d^	361	29.18	1.32 (1.16-1.50)	1.41 (1.23-1.60)	1.39 (1.22-1.58)
Depressive symptoms scores, quintile^e^					
1 (0-3)	244	18.52	1 [Reference]	1 [Reference]	1 [Reference]
2 (4-6)	205	20.41	1.08 (0.89-1.29)	1.12 (0.93-1.35)	1.08 (0.90-1.31)
3 (7-9)	162	20.35	1.05 (0.86-1.28)	1.10 (0.90-1.35)	1.10 (0.90-1.34)
4 (10-14)	224	25.19	1.27 (1.06-1.52)	1.37 (1.14-1.65)	1.34 (1.11-1.62)
5 (15-30)	253	33.02	1.62 (1.35-1.93)	1.82 (1.52-2.19)	1.75 (1.45-2.11)
Heart disease					
Depressive symptoms status					
No symptoms	622	17.59	1 [Reference]	1 [Reference]	1 [Reference]
Symptoms^d^	307	24.82	1.29 (1.13-1.49)	1.39 (1.21-1.60)	1.36 (1.18-1.57)
Depressive symptoms scores, quintile^e^					
1 (0-3)	205	15.56	1 [Reference]	1 [Reference]	1 [Reference]
2 (4-6)	174	17.32	1.08 (0.88-1.32)	1.13 (0.93-1.39)	1.10 (0.89-1.34)
3 (7-9)	144	18.09	1.10 (0.89-1.36)	1.17 (0.95-1.45)	1.15 (0.93-1.43)
4 (10-14)	191	21.48	1.27 (1.04-1.55)	1.38 (1.13-1.69)	1.35 (1.10-1.65)
5 (15-30)	215	28.06	1.60 (1.32-1.94)	1.83 (1.50-2.23)	1.73 (1.42-2.12)
Stroke					
Depressive symptoms status					
No symptoms	125	20.55	1 [Reference]	1 [Reference]	1 [Reference]
Symptoms^d^	65	29.18	1.49 (1.10-2.03)	1.45 (1.06-1.98)	1.45 (1.06-1.99)
Depressive symptoms scores, quintile^e^					
1 (0-3)	43	18.52	1 [Reference]	1 [Reference]	1 [Reference]
2 (4-6)	37	20.41	1.13 (0.73-1.75)	1.13 (0.72-1.75)	1.11 (0.71-1.72)
3 (7-9)	25	20.35	0.95 (0.58-1.55)	0.94 (0.57-1.54)	0.96 (0.58-1.58)
4 (10-14)	41	25.19	1.43 (0.93-2.19)	1.39 (0.90-2.15)	1.39 (0.90-2.16)
5 (15-30)	44	33.02	1.78 (1.16-2.72)	1.71 (1.11-2.66)	1.69 (1.09-2.64)

Abbreviation: HR, hazard ratio.

^a^ Model 1 was adjusted for age and sex.

^b^ Model 2 was adjusted for age, sex, residence, marital status, educational level, smoking status, and drinking status.

^c^ Model 3 was adjusted as model 2 plus systolic blood pressure and body mass index; history of diabetes, hypertension, dyslipidemia, and chronic kidney disease; and use hypertension medications, diabetes medications, and lipid-lowering therapy.

^d^ Defined as a score of 12 or greater on the 10-item Center for Epidemiologic Studies Depression Scale.

^e^ The depressive symptoms score, measured by the 10-item Center for Epidemiologic Studies Depression Scale, varies between 0 to 30, with the highest score representing the highest risk of depressive symptoms.

Of the individual depressive symptoms, the most common ones included feeling hopeless (35.9%), restless sleep (32.7%), and being bothered by little things (31.7%) (Table 3). Table 3 shows the associations of individual depressive symptoms and incident CVD events. When entering all 10 individual depressive symptoms as measured by the CES-D items in model 3, only 2 symptoms (sleep was restless: adjusted HR, 1.21; 95% CI, 1.06-1.39; and felt lonely: adjusted HR, 1.21; 95% CI, 1.02-1.44) were significantly associated with incident CVD, after adjusting for potential confounders. Feeling lonely was also significantly associated with stroke (HR, 2.10; 95% CI, 1.43-3.10).

**Table 3.** Association Between Specific Depressive Symptoms and Cardiovascular Diseases

Items^a^	Symptom, No. (%)	HR (95% CI)^b^		
		Cardiovascular Disease	Heart Disease	Stroke
Bothered by little things	3936 (31.7)	1.14 (0.98-1.32)	1.15 (0.98-1.36)	1.11 (0.77-1.59)
Had trouble concentrating	3469 (27.9)	0.93 (0.80-1.08)	0.95 (0.81-1.11)	0.78 (1.54-1.13)
Felt depressed	3633 (29.3)	1.05 (0.89-1.23)	1.13 (0.95-1.35)	0.61 (0.40-0.91)
Everything was an effort	3807 (30.7)	0.94 (0.81-1.09)	0.91 (0.77-1.08)	1.03 (0.72-1.48)
Did not feel hopeful	4461 (35.9)	1.09 (0.95-1.24)	1.08 (0.93-1.24)	1.22 (0.89-1.67)
Felt fearful	1184 (9.5)	1.10 (0.90-1.35)	1.14 (0.92-1.41)	0.97 (0.60-1.57)
Sleep was restless	4063 (32.7)	1.21 (1.06-1.39)	1.16 (1.01-1.34)	1.48 (1.08-2.04)
Did not feel happy	3690 (29.7)	1.11 (0.96-1.28)	1.10 (0.94-1.29)	1.11 (0.79-1.57)
Felt lonely	1926 (15.5)	1.21 (1.02-1.44)	1.09 (0.90-1.32)	2.10 (1.43-3.10)
Could not get going	1205 (9.7)	1.08 (0.88-1.33)	1.03 (0.83-1.29)	1.35 (0.86-2.12)

Abbreviation: HR, hazard ratio.

^a^ Measured by the 10-item Center for Epidemiologic Studies Depression Scale.

^b^ Model was adjusted for the 10 items of individual depressive symptoms, age, sex, residence, marital status, educational level, smoking status, drinking status, systolic blood pressure, and body mass index; history of diabetes, hypertension, dyslipidemia, and chronic kidney disease; and use of hypertension medications, diabetes medications, and lipid-lowering therapy.

**Figure 2.**
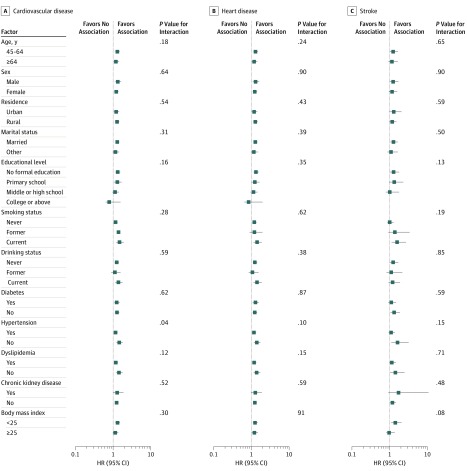
Association Between Depressive Symptoms and Cardiovascular Disease Events Risk Stratified by Different Factors Graphs show hazard ratios (HRs) and 95% CIs for cardiovascular disease (A), heart disease (B), and stroke (C) adjusted for age, sex, residence, marital status, educational level, smoking status, drinking status, and body mass index (calculated as the weight in kilograms divided by height in meters squared); and history of diabetes, hypertension, dyslipidemia, chronic kidney disease.

Figure 2 shows the association between depressive symptoms and incident CVD events stratified by potential risk factors. The association between depressive symptoms and incident CVD was more pronounced among participants without hypertension (adjusted HR, 1.68; 95% CI, 1.36-2.07), compared with those with hypertension (adjusted HR, 1.27; 95% CI, 1.07-1.50) at baseline (*P* = .04 for interaction). The results did not significantly change after further adjusting for total cholesterol, triglycerides, high-density lipoprotein cholesterol, low-density lipoprotein cholesterol, fasting plasma glucose, estimated glomerular filtration rate, or high-sensitivity C-reactive protein levels (eTable 2 in the Supplement). Similar results were found when complete data analyses were conducted (eTable 3 in the Supplement). In addition, elevated depressive symptoms were significantly associated with 1.41-fold (95% CI, 1.23-1.62) incident CVD when using the Fine and Gray model with death as competing risk event (eTable 4 in the Supplement).

## Discussion

This study examined the associations between depressive symptoms and incident CVD in a nationally representative cohort of 12 417 adults in China aged 45 years and older with over 4 years of follow-up. At baseline, 26.0% of the participants experienced depressive symptoms. Depressive symptoms were associated with a 39.0% increased risk of CVD. The presence of certain depressive symptoms (restless sleep and loneliness) were independently associated with incident CVD.

Increasing evidence suggests that the presence of depressive symptoms is associated with an increased risk of CVD.^8,10,33,34,35^ The Jackson Heart Study^20^ found an almost 2-fold increase in the risk of coronary heart disease and stroke in patients with major depression. The Reasons for Geographical and Racial Differences in Stroke Study^36^ also found that severe depressive symptoms were associated with an increased risk of coronary heart disease, stroke, and mortality. A recent cohort study^22^ suggested that time-dependent depressive symptoms were associated with 1.4-fold risk of cardiovascular mortality. In addition, several meta-analyses and systematic reviews^12,13,21^ found a positive association between severe depressive symptoms and increased risk of CVD. As expected, the risk of CVD including heart disease and stroke was associated with depressive symptoms in this study.

Although the association between depressive symptoms and incident CVD has been widely examined, the contribution of specific depressive symptoms to incident cardiovascular events is still not clear. This study found that restless sleep and loneliness were independently associated with incident CVD and stroke. Restless sleep or insomnia previously have been associated with CVD.^37,38,39^ In the Health and Retirement Study Cohort,^8^ 2 individual depressive symptoms (everything was an effort and restless sleep) were independently associated with an increased risk of stroke. A recent study^9^ also found that a combination of depressive symptoms and sleep problems was associated with an increase in the odds of coronary heart disease at 6-year follow-up. The mechanisms that underlie the association between sleep problems and increased risk of CVD have been widely examined.^38^ Some studies found that short sleep duration or nonrestorative sleep could lead to metabolic or endocrine changes through elevated levels of inflammatory cytokines and sympathetic activation,^40,41^ poor sleep quality with low levels of slow-wave sleep could impair glucose tolerance and then increase the risk of type 2 diabetes,^42^ and sleep curtailment could increase cortisol secretion and alter circulating levels of growth hormone, leptin, and ghrelin,^43^ all of which are associated with an increased risk of CVD.

In the cross-sectional Netherlands Study of Depressed Older Persons, Hegeman et al^44^ found an independent association between loneliness and CVD in women (odds ratio, 1.13; 95% CI, 1.06-1.21). On the basis of a secondary analysis of the English Longitudinal Study of Aging, Valtorta et al^45^ found that loneliness was associated with a 1.27-fold increased risk of CVD (HR, 1.27; 95% CI, 1.01-1.57). Loneliness could increase the activity of the hypothalamic-pituitary-adrenal axis, leading to higher levels of circulating cortisol.^46,47,48^ Increased cortisol levels further affect blood pressure and vascular endothelial function through the vascular nitric oxide system,^49^ which may account for the association between loneliness and CVD. However, 2 longitudinal studies^50,51,52^ of older adults in the United States and United Kingdom reported that loneliness did not have cardiometabolic effects. Apart from differences in methods, we assume that different sociocultural contexts across these countries may partly explain the different findings between studies,^51,52^ although convincing findings are needed to support this notion. In China, loneliness among elders may take on a specific relevance. As China continues to develop, it is less common for young people to comport with traditional Confucian ideals of filial piety, and this, coupled with massive internal migration, may disruptive to care structures for older adults.^53^

The underlying mechanisms of the association between depressive symptoms and CVD are multifactorial, involving autonomic nerve dysfunction, inflammation, endothelial dysfunction, platelet activation and thrombosis, life behavior, and cardiac metabolic risk factors.^54^ For example, a meta-analysis^55^ found that depression may be associated with dietary patterns, which could change the gut microbiome and then increase CVD risk. Furthermore, the association between elevated levels of inflammatory markers, such as high-sensitivity C-reactive protein, and depression are well documented.^56,57^ In the current study, after adjusting for high-sensitivity C-reactive protein, the association between depressive symptoms and increased risk of CVD remained, which suggests these findings are robust. In this study, apart from restless sleep and loneliness, other individual depressive symptoms as measured by the CES-D were not significantly associated with incident CVD. We have no clear explanation about the associations between different individual depressive symptoms and incident CVD except for assuming that the effect size between both restless sleep and loneliness and CVD is, perhaps, greater than those for other individual depressive symptoms because of biological and environmental factors.^38,49^ In addition, the associations between other depressive symptoms and CVD may be undetected because of the short study period (4 years).

### Strengths and Limitations

The strengths of this study included the prospective design, the long follow-up period, and the inclusion of specific depressive symptoms. However, several limitations need to be addressed. First, some confounding factors of the association between depression and CVD, such as income, social support, isolation, and joblessness,^58,59^ were not adjusted in this study. Second, similar to other studies,^60,61^ for logistical reasons, the diagnosis of CVD was self-reported. Medical records were not available in the CHARLS; however, some other large-scale studies,^28^ such as the English Longitudinal Study of Aging, found that self-reported incident coronary heart disease had a good agreement with medical records (accuracy, 77.5%). Third, only participants from China were involved in this study; thus the findings may not fully generalize to other countries. In addition, time-varying exposures were not included in the present analysis, so residual confounding is a concern.

## Conclusions

This study found that the presence of certain depressive symptoms, such as restless sleep and loneliness, could be associated with an increased risk of CVD in middle-aged and older Chinese adults. To reduce the risk of CVD, effective treatment and psychosocial interventions should be delivered targeting these symptoms.

## Notes

Supplement. **eTable 1.** Baseline Characteristics Between Participants Included and Not Included **eTable 2.** Association of Depressive Symptoms With Cardiovascular Diseases in Subpopulations of 8696 Participants With Metabolic Biomarkers Measurements **eTable 3.** Association of Depressive Symptoms With Cardiovascular Diseases in Subpopulations of 10 186 Participants With Complete Data **eTable 4.** Association of Depressive Symptoms With Cardiovascular Diseases by Competing Risk Analysis
