# Errors in Results, Table 3, and Figure 2

**DOI:** 10.1001/jamanetworkopen.2019.20603

**Published:** 2020-01-22

In the Original Investigation titled “Association of Depressive Symptoms With Incident Cardiovascular Diseases in Middle-Aged and Older Chinese Adults,”^1^ published December 4, 2019, there was an error in the first sentence of the Results section, where the number 1841 should have appeared as 484. In the second paragraph of the Results, fifth sentence, the number 19.91 should have appeared as 1.91, and in the final sentence of that paragraph, *P* < .02 should have appeared as *P* = .02. In Table 3 in the second row of the Stroke column, the 95% CI for 0.78 should have appeared as 0.54-1.13. In Figure 2A, the older age group should have appeared as ≥65 and the *P* values for residence and drinking status both should have appeared as .56. In Figure 2B, the *P* value for body mass index should have appeared as .91. This article has been corrected.^1^
