# Errors in Discussion and Table 2

**DOI:** 10.1001/jamanetworkopen.2020.1449

**Published:** 2020-02-28

In the Original Investigation titled “Association of Depressive Symptoms With Incident Cardiovascular Diseases in Middle-Aged and Older Chinese Adults,”^1^ published December 4, 2019, there was an error in the Strengths and Limitations subsection of the Discussion. The first sentence should read, “The strengths of this study included the prospective design and the inclusion of specific depressive symptoms.” There were also errors in Table 2. In the third column, under cardiovascular disease, the value for no depressive symptoms should be 20.56. In the same column, under stroke, the value for no depressive symptoms should be 3.53, the value for depressive symptoms should be 5.25, and the values for depressive symptom score quintiles should be 3.26, 3.68, 3.14, 4.61, and 5.74, respectively. This article has been corrected.^1^
