# Supplementary material for: Association of Depressive Symptoms With Incident Cardiovascular Diseases in Middle-Aged and Older Chinese Adults
Source: JAMA Netw Open. 2019 Dec 4;2(12):e1916591. doi: 10.1001/jamanetworkopen.2019.16591 (PMC6902756; doi:10.1001/jamanetworkopen.2019.16591)
Supplement: Supplement. — eTable 1. Baseline Characteristics Between Participants Included and Not Included eTable 2. Association of Depressive Symptoms With Cardiovascular Diseases in Subpopulations of 8696 Participants With Metabolic Biomarkers Measurements eTable 3. Association of Depressive Symptoms With Cardiovascular Diseases in Subpopulations of 10 186 Participants With Complete Data eTable 4. Association of Depressive Symptoms With Cardiovascular Diseases by Competing Risk Analysis [file jamanetwopen-2-e1916591-s001.pdf]

# Supplementary Online Content

Li H, Zheng D, Li Z, et al. Association of depressive symptoms with incident cardiovascular diseases in middle-aged and older Chinese adults. *JAMA Netw Open*. 2019;2(12):e1916591. doi:10.1001/jamanetworkopen.2019.16591

**eTable 1.** Baseline Characteristics Between Participants Included and Not Included

**eTable 2.** Association of Depressive Symptoms With Cardiovascular Diseases in Subpopulations of 8696 Participants With Metabolic Biomarkers Measurements

**eTable 3.** Association of Depressive Symptoms With Cardiovascular Diseases in Subpopulations of 10 186 Participants With Complete Data

**eTable 4.** Association of Depressive Symptoms With Cardiovascular Diseases by Competing Risk Analysis

This supplementary material has been provided by the authors to give readers additional information about their work.

© 2019 Li H et al. *JAMA Network Open.*

**eTable 1. Baseline Characteristics Between Participants Included and Not Included**

| **Characteristics** | **Excluded** | **Included** | ***P* Value** |
|---|---|---|---|
| No. of participants (%) | 5291 (29.9) | 12417 (70.1) | |
| Age, y, mean (SD) | 59.37 (11.49) | 58.40 (9.51) | <.001 |
| Men, No. (%) | 2363 (44.7) | 6113 (49.2) | <.001 |
| Rural residence, No. (%) | 2960 (55.9) | 7577 (61.0) | <.001 |
| Married, No. (%) | 3826 (72.8) | 10344 (83.3) | <.001 |
| Educational level, No. (%) | | | 0.05 |
| No formal education | 2441 (46.3) | 5508 (44.4) | |
| Primary school | 1127 (21.4) | 2695 (21.7) | |
| Middle or high school | 1455 (27.6) | 3648 (29.4) | |
| College or above | 252 (4.8) | 565 (4.6) | |
| Smoking status, No. (%) | | | <.001 |
| Never | 3205 (71.3) | 7414 (59.7) | |
| Former | 406 (9.0) | 1011 (8.1) | |
| Current | 882 (19.6) | 3989 (32.1) | |
| Drinking status, No. (%) | | | <.001 |
| Never | 3137 (61.2) | 7196 (58.0) | |
| Former | 490 (9.6) | 953 (7.7) | |
| Current | 1502 (29.3) | 4265 (34.4) | |
| History of diabetes, No. (%) | 481 (9.5) | 628 (5.1) | <.001 |
| History of hypertension, No. (%) | 1870 (36.6) | 2768 (22.4) | <.001 |
| History of dyslipidemia, No. (%) | 741 (14.8) | 960 (7.9) | <.001 |
| History of CKD, No. (%) | 427 (8.4) | 629 (5.1) | <.001 |
| Diabetes medications, No. (%) | 320 (6.3) | 392 (3.2) | <.001 |
| Hypertension medications, No. (%) | 1468 (28.7) | 1946 (15.7) | <.001 |
| Lipid-lowering therapy, No. (%) | 449 (9.0) | 464 (3.8) | <.001 |
| SBP, mean (SD), mm Hg | 132.72 (22.76) | 130.15 (21.31) | <.001 |
| DBP, mean (SD), mm Hg | 76.29 (12.58) | 75.89 (12.13) | 0.10 |
| BMI, kg/m$^2$, mean (SD) | 23.90 (4.18) | 23.34 (3.85) | <.001 |
| Abbreviations: CKD, chronic kidney disease; SBP, systolic blood pressure; DBP, diastolic blood pressure; BMI, body mass index. | | | |

© 2019 Li H et al. *JAMA Network Open.*

**eTable 2. Association of Depressive Symptoms With Cardiovascular Diseases in Subpopulations of 8696 Participants With Metabolic Biomarkers Measurements**

| Model | HR (95% CI) | | |
|---|---|---|---|
| | **Cardiovascular disease** | **Heart disease** | **Stroke** |
| Model 3 [a] | 1.36 (1.17-1.58) | 1.30 (1.11-1.54) | 1.54 (1.06-2.23) |
| Model adjusted as model 3 plus | | | |
| Total Cholesterol | 1.39 (1.19-1.62) | 1.33 (1.13-1.57) | 1.54 (1.06-2.23) |
| Triglycerides | 1.39 (1.19-1.62) | 1.33 (1.13-1.57) | 1.54 (1.06-2.23) |
| HDL cholesterol | 1.39 (1.19-1.61) | 1.33 (1.13-1.57) | 1.54 (1.06-2.23) |
| LDL cholesterol | 1.38 (1.19-1.61) | 1.33 (1.13-1.57) | 1.54 (1.06-2.23) |
| FPG | 1.39 (1.19-1.62) | 1.34 (1.13-1.57) | 1.54 (1.06-2.23) |
| eGFR | 1.39 (1.19-1.62) | 1.34 (1.13-1.57) | 1.54 (1.06-2.23) |
| hs-CRP | 1.39 (1.19-1.61) | 1.33 (1.13-1.57) | 1.53 (1.06-2.24) |
| All biomarkers | 1.38 (1.19-1.61) | 1.33 (1.13-1.57) | 1.53 (1.06-2.22) |

Abbreviations: HR, hazard ratio; HDL, high-density lipoprotein; LDL, low-density lipoprotein; FPG, fasting plasma glucose; eGFR, estimated glomerular filtration rate; hs-CRP, high-sensitivity C-reactive protein.

[e] Model 3 adjusted for age, sex, residence, marital status, educational level, smoking status, drinking status， history of diabetes, hypertension, dyslipidemia, and chronic kidney disease, and hypertension medications, diabetes medications, lipid-lowering therapy, systolic blood pressure, and body mass index.

© 2019 Li H et al. *JAMA Network Open.*

**eTable 3. Association of Depressive Symptoms With Cardiovascular Diseases in Subpopulations of 10 186 Participants With Complete Data**

| Model | HR (95% CI) | | |
|---|---|---|---|
| | **Cardiovascular disease** | **Heart disease** | **Stroke** |
| Model 1 [a] | 1.36 (1.18-1.57) | 1.31 (1.12-1.52) | 1.66 (1.18-2.23) |
| Model 2 [b] | 1.45 (1.25-1.67) | 1.41 (1.21-1.65) | 1.62 (1.14-2.29) |
| Model 3 [c] | 1.44 (1.24-1.66) | 1.39 (1.19-1.63) | 1.61 (1.13-2.29) |

Abbreviations: HR, hazard ratio.

[a] Model 1 adjusted for age and sex.

[b] Model 2 adjusted for age, sex, residence, marital status, educational level, smoking status, and drinking status.

[c] Model 3 adjusted as model 2 plus history of diabetes, hypertension, dyslipidemia, and chronic kidney disease, and hypertension medications, diabetes medications, lipid-lowering therapy, systolic blood pressure, and body mass index.

© 2019 Li H et al. *JAMA Network Open*.

**eTable 4. Association of Depressive Symptoms With Cardiovascular Diseases by Competing Risk Analysis**

| Model | HR (95% CI) [a] | | |
|---|---|---|---|
| | **Cardiovascular disease** | **Heart disease** | **Stroke** |
| Model 1 [b] | 1.31 (1.16-1.48) | 1.27 (1.11-1.46) | 1.56 (1.15-2.11) |
| Model 2 [c] | 1.38 (1.21-1.56) | 1.35 (1.18-1.55) | 1.51 (1.11-2.06) |
| Model 3 [d] | 1.41 (1.23-1.62) | 1.37 (1.18-1.60) | 1.66 (1.16-2.36) |

Abbreviations: HR, hazard ratio.

[a] HR was estimated from Fine and Gray model.

[c] Model 1 adjusted for age and sex.

[d] Model 2 adjusted for age, sex, residence, marital status, educational level, smoking status, and drinking status.

[e] Model 3 adjusted as model 2 plus history of diabetes, hypertension, dyslipidemia, and chronic kidney disease, and hypertension medications, diabetes medications, lipid-lowering therapy, systolic blood pressure, and body mass index.

© 2019 Li H et al. *JAMA Network Open.*
